# Weed and Corn Seedling Detection in Field Based on Multi Feature Fusion and Support Vector Machine

**DOI:** 10.3390/s21010212

**Published:** 2020-12-31

**Authors:** Yajun Chen, Zhangnan Wu, Bo Zhao, Caixia Fan, Shuwei Shi

**Affiliations:** 1 Department of Information Science, Xi’an University of Technology, Xi’an 710048, China; 2190820003@stu.xaut.edu.cn (Z.W.); fcx1981@xaut.edu.cn (C.F.); 2 Chinese Academy of Agricultural Mechanization Sciences, Beijing 100083, China; zhaoboshi@126.com; 3 Zhengzhou Cotton & Jute Engineering Technology and Design Research Institute, Zhengzhou 451162, China; shishuwei88@163.com

**Keywords:** precision spraying, precise fertilization, multi-feature, weed and corn seedling detection, support vector machine, rotation invariant LBP, Gabor feature, co-occurrence matrix

## Abstract

Detection of weeds and crops is the key step for precision spraying using the spraying herbicide robot and precise fertilization for the agriculture machine in the field. On the basis of k-mean clustering image segmentation using color information and connected region analysis, a method combining multi feature fusion and support vector machine (SVM) was proposed to identify and detect the position of corn seedlings and weeds, to reduce the harm of weeds on corn growth, and to achieve accurate fertilization, thereby realizing precise weeding or fertilizing. First, the image dataset for weed and corn seedling classification in the corn seedling stage was established. Second, many different features of corn seedlings and weeds were extracted, and dimensionality was reduced by principal component analysis, including the histogram of oriented gradient feature, rotation invariant local binary pattern (LBP) feature, Hu invariant moment feature, Gabor feature, gray level co-occurrence matrix, and gray level-gradient co-occurrence matrix. Then, the classifier training based on SVM was conducted to obtain the recognition model for corn seedlings and weeds. The comprehensive recognition performance of single feature or different fusion strategies for six features is compared and analyzed, and the optimal feature fusion strategy is obtained. Finally, by utilizing the actual corn seedling field images, the proposed weed and corn seedling detection method effect was tested. LAB color space and K-means clustering were used to achieve image segmentation. Connected component analysis was adopted to remove small objects. The previously trained recognition model was utilized to identify and label each connected region to identify and detect weeds and corn seedlings. The experimental results showed that the fusion feature combination of rotation invariant LBP feature and gray level-gradient co-occurrence matrix based on SVM classifier obtained the highest classification accuracy and accurately detected all kinds of weeds and corn seedlings. It provided information on weed and crop positions to the spraying herbicide robot for accurate spraying or to the precise fertilization machine for accurate fertilizing.

## 1. Introduction

Corn is among the most important cereal crops in China. At present, the main weeding methods in corn fields include artificial, mechanical, and chemical weeding. Chemical weeding has the benefit of being low cost, and there is no need to care about the terrain. Thus, it is widely used at home and abroad. However, the problem with chemical weeding is the full coverage field spraying of herbicides without distinguishing between crops and weeds, which causes much herbicide waste, increases pollution, and increases soil dependence on chemical agents [1]. With the maturity and improvement of image processing, machine vision technology, and the development needs of precision agriculture, countries have begun to study the use of computer vision technology to achieve the precise use of herbicides [2,3,4]. In addition, precise fertilization has also become a trend. It is very important to accurately locate weeds and crop seedlings in the field.

In recent years, deep learning has been extended to agriculture [5]. However, considering that traditional learning methods have lower requirements for the graphics processing unit, can be deployed in agricultural machinery equipment at lower costs, and require a small sample size and short training time, many scholars are conducting further studies on the use of traditional methods to improve the accuracy of weed detection. In the early stage, many scholars at home and abroad used shape features, such as second moment, contrast, entropy or roundness, rectangularity, eccentricity, aspect ratio, and other shape features [6,7,8] or texture features [9] of leaves to identify crops or weeds. For example, Ishak et al. used Gabor features and gradient field distribution to realize weed classification [10]. Naresh et al. used the improved local binary pattern to identify different plant leaves [11]. Le et al. determined the distinction between corn and single weed [12]. Ma and other researchers used the histogram of oriented gradient (HOG) feature and support vector machine (SVM) to identify grape leaves, which are robust to light and environmental changes, but they could not solve the problem of grape leaf detection with incorrect posture [13]. Some scholars also used plant density and location information to improve the accuracy of recognition [14,15,16], but these methods are easily affected by vibration, gyro, or uncontrolled motion in actual application [17]. In general, these studies provided effective methods and approaches for early plant recognition and weed detection based on image technology. However, many studies focused on the identification of plants using single features only for the identification of different plant leaves and not to accurately detect crops or weeds in the field. Moreover, some results showed poor identification, low accuracy, and low stability.

To deal with the problems of low accuracy and poor stability of a single feature in the field, some scholars proposed the fusing of multiple features to further improve the accuracy of identification [18]. For example, Mao and others achieved the segmentation and recognition of soil, wheat seedlings, and weeds based on color and texture features [19]. Tang et al. used improved local binary pattern (LBP) and gray level co-occurrence matrix (GLCM) to classify tea [20]. Chowdhury et al. combined texture information with color information; GLCM and color features were combined to extract feature data to identify roadside weeds, and good recognition and classification results were obtained [21]. He et al. fused the multi-source recognition information of different features, such as shape, fractal dimension, and texture, and proposed an optimization method of SVM combined with D-S evidence theory, which further improved the accuracy and stability of weed identification [22]. Liu et al. [23] combined LBP, Hu moment invariants, Gabor, GLCM, Fourier descriptors, and other features with deep belief network (DBN) to realize plant leaf recognition. Chaki et al. combined Gabor and GLCM features and achieved the leaf recognition of 31 species of plants using multilayer perceptron [24]. Bakhshipour et al. extracted three shape features for sugar beet and weeds and combined them with SVM for detection. This method is also effective in the case of low leaf occlusion and overlap [25]. However, most of these studies only aimed to develop identification methods of different plant leaf images and were not focused on the identification and location of plants and weeds in a farmland. The application of weed identification and detection in an actual farmland still needs further research.

Among the traditional methods, most relevant scholars use SVM and artificial neural network (ANN) to solve weed recognition and detection task, both of which can achieve the purpose of weed recognition. SVM is more accurate than ANN. For example, Adel Bakhshipour et al. (2018) evaluated the application of shape featured-based SVMs and ANNs in weed detection [25], and the overall classification accuracy of SVM was 2.08% higher than that of ANN. Therefore, in the present study, we used SVM classifier to identify weeds and corn seedlings.

The methods based on multiple features of plant leaf recognition and other field crop identification research can serve as references for field weed and crop identification and detection research in the corn seedling stage. In addition, some scholars did not specifically analyze which feature descriptor was more suitable for the target feature extraction when using the shape, color, and texture features of the target. The comparison of the specific feature descriptor selection schemes is lacking. For example, LBP, GLCM, gray level-gradient co-occurrence matrix (GGCM), and Gabor are commonly used in texture feature description. In a complex field environment, accuracy, timeliness, and stability still require specific analysis. The main contributions of this paper are as follows. Two small image datasets for weed and corn seedling classification and detection in corn seedling stage were established. One was the leaf dataset used to train and verify the leaf classification models of corns and weeds, including 1000 positive sample sets (corn leaves) and 1000 negative sample sets (weeds). The other was the actual field image set for testing, in which 400 actual field images containing corn seedling and weeds were present. Second, the problem of weed and corn seedling detection was transformed into the problem of binary classification and object detection of weeds and corn seedlings. At the same time, comprehensive experiments of weed detection were conducted under the conditions of HOG, rotation invariant LBP, Hu invariant moment, Gabor, GGCM, GLCM, and their different feature fusion strategies. These six features are the most commonly used feature descriptors in plant leaf recognition in recent years. A feature fusion scheme with relatively high classification accuracy was proposed, and the position detection of corn seedlings and weeds was achieved based on k-mean clustering image segmentation using color information and connected region analysis and SVM classifier, which provided location information to the spraying pesticide robot for the accurate spraying of weeds or to the precise fertilizer machine in later stages.

## 2. Materials and Methods

### 2.1. Basic Idea of Experiment

**Figure 1:**
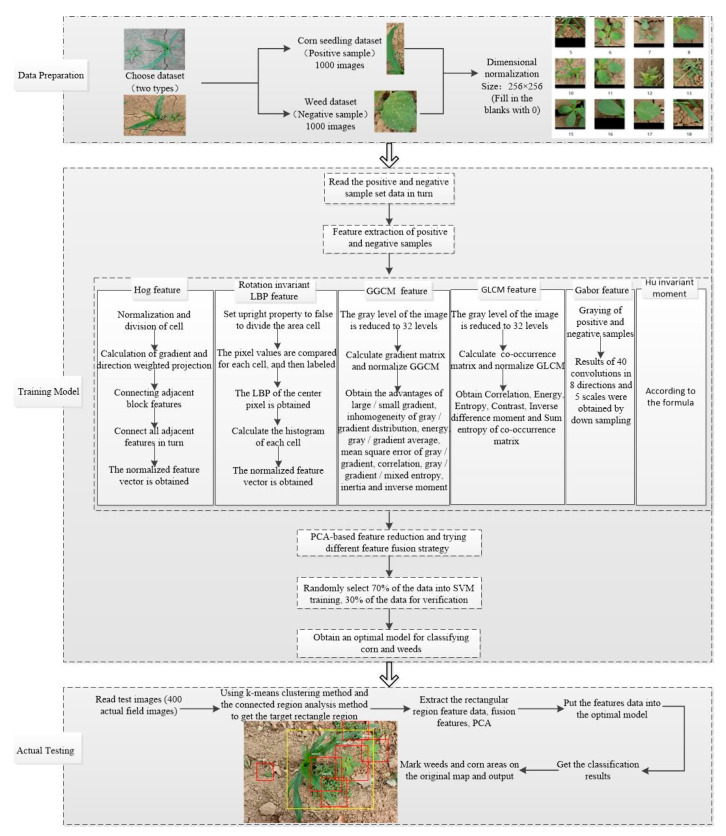
The basic idea flow chart of the experiment.

A method that combines multi-feature fusion with SVM to automatically identify corn seedlings and weeds is proposed. The method can acquire the respective positions of corn seedlings and weeds. The experimental results were obtained by comparing different fusion strategies of feature descriptors, and the optimal fusion strategy for weed or corn classification was obtained. The feature layer fusion scheme was used to achieve the highest classification and detection accuracy of corn seedlings and weeds based on the SVM classification method. The basic idea and method flow chart of this paper is shown in Figure 1. Figure 1 describes the weed and corn seedling detection method based on multi-feature fusion and SVM from data preparation, training model, and actual test in an abstract manner. The first part was data preparation. A small database for weed and corn seedling classification at corn seedling stage was established, and the positive and negative sample datasets and actual field test images required for training were established. Detailed image preprocessing was performed (Section 2.2). The second part was the training model stage. A comprehensive experiment was conducted for weed and corn seedling classification under six features including HOG, rotation invariant LBP, HU invariant moment, Gabor, GGCM, and GLCM with different fusion strategies. Parameter setting and dimension description of each feature distributed are mentioned in Section 2.3, and specific training steps are presented in Section 2.4.1. The third part is the actual testing stage, which used 400 actual field images to test and observe the performance of the proposed weed and corn seedling detection algorithm in a real complex background. The specific test process is in Section 2.4.2, and the test result is in Section 3.2.

### 2.2. Dataset Establishment and Preprocessing

**Figure 2:**
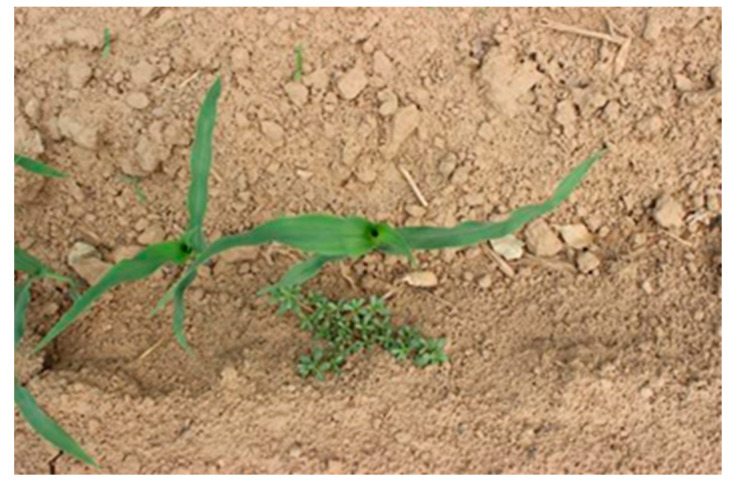
Images taken on sunny days.

**Figure 3:**
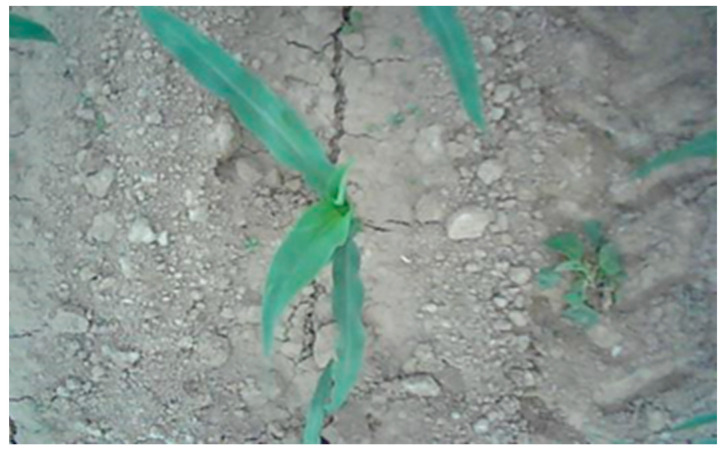
Images taken on cloudy days.

The experimental images were obtained from the Chinese Academy of Agricultural Mechanization Sciences. The images of 2–5 leaves of corn and weeds in the seedling stage were collected under different periods of natural light. The dataset relied on array cameras mounted on agricultural machinery to continuously capture top-down images. To increase the complexity of the sample, two kinds of dataset mixed mode were selected. The first type comprised the corn field images taken on sunny days. The second type comprised the images taken on cloudy days, as shown in Figure 2 and Figure 3. The collected images partially overlapped due to the use of the array camera. So, some similar images were present in the original collection of images. To make the training process effective, images without weeds or corn targets and almost repeated images were eliminated. We tried our best to make the dataset as typical as possible. Based on these images, the algorithm test dataset was established.

Our collected images included four kinds of associated weeds common in early corn fields, namely, *Cirsium setosum* (Willd.) MB, *Poa annua* L., *Eleusine indica* (L.) Gaertn., and *Chenopodium album* L. Corn leaves were in the 2–5 leaf stage. The actual weeds growing in the field were natural and random and were in the germination, seedling, and vegetative growth stages, among which the weeds in the latter two stages were in the majority.

**Figure 4:**
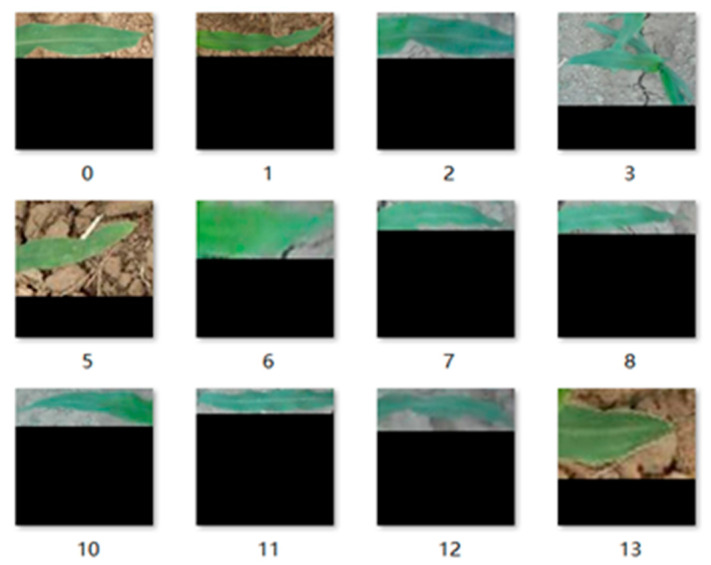
Positive sample dataset.

**Figure 5:**
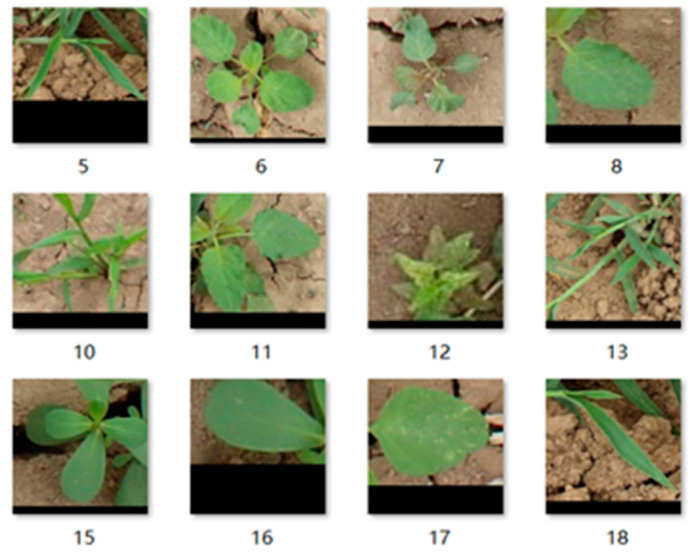
Negative sample dataset.

First, we selected a part of the actual field images. Then, we cropped the regions of the corn and weed leaves in the image using the Image Labeler tool and automatically cut out the leaf region. To contain the complete leaf information as much as possible, only the complete leaves in the image were intercepted. The images of corn seedling and weed leaves were taken as positive and negative samples, respectively. Figure 4 shows the positive sample data after size normalization, and Figure 5 shows the negative sample data after size normalization. To facilitate the test, 1000 positive and negative samples were selected.

In the process of establishing the first dataset for weed and corn classification, to ensure the implementation of various feature extraction algorithms in the follow-up research, it is necessary to normalize the image size. To address the problem of image size normalization, many researchers generally did not consider the shape features of the object in the image, and then, the size normalization was conducted by coarse scaling directly. In this paper, we proposed a strategy to keep the shape of leaves unchanged and supplement the blank region of normalized size with pixel 0. We tested the two strategies in the experiment, and the classification accuracy of weeds and maize seedlings using our size normalization strategy was higher. Therefore, the size of all images in the positive and negative sample set was adjusted to 256×256 pixels, and the blank region was filled with 0 in the process of size normalization to ensure that the leaf shape of corn seedlings and weeds remains unchanged. Thus, a small image dataset for weed and corn seedling recognition algorithm was constructed. In addition, the actual field test image dataset was established from the remaining original field images, which contained 400 actual corn seedling field images with weeds. The images were selected to test the actual weed detection effect. The number of data sets and image size in the research is shown in Table 1.

### 2.3. Feature Extraction of Crops and Weeds

The texture feature is a regional feature reflecting the spatial distribution of pixels. The shape of corn leaves is flat and contains much texture information; it belongs to the combination of regular and random textures [26]. In this paper, GLCM and GGCM based on statistical texture analysis, rotation invariant LBP based on structural texture analysis, and Gabor features based on signal processing analysis were selected. In addition to these four texture features, HOG and Hu moment invariants based on shape features were also used. The above six feature descriptors were fused to form 18 feature combinations (6 groups of single features, 12 groups of double feature fusion, 3 groups of three feature fusion, 2 groups of four feature fusion, and 1 group of five feature fusion). Principal component analysis (PCA) was used to reduce the dimension of features with more dimensions, and some new information was extracted from the original data, which reduced the number of variables but also attained the main contradiction. In the experiment, the number of principal component retention also affected the final experimental accuracy. After multiple retention tests, the principal component retention number with the highest accuracy was selected as the feature dimension of the descriptor, and the subsequent multi feature fusion was conducted on this basis. After dimensionality reduction, HOG features were 55 dimensions, rotation invariant LBP features were 95 dimensions, and Gabor features were 360 dimensions. The numbers of six GLCM feature parameters and 15 GGCM feature parameters were small, and they did not need to be involved in dimension reduction. Under 18 sets of multi feature fusion strategies, the combination of Hog and Gabor features had the largest dimension, and the dimension was 684.

#### 2.3.1. HOG Features

HOG feature, a feature descriptor for target detection, was first proposed in 2005. Its main principle is to use gradient or directional density of edge to describe the local contour of the object in the image, which has strong robustness to the changes of illumination and background under natural conditions [13]. When extracting HOG features, 8×8, 16×16, 32×32, 64×64, and 128×128 cell size were selected to divide the image. Then, the feature dimensions of the whole image were 34,596, 8100, 1764, 324, and 36 dimensions, respectively. The experimental results of different dimensions were shown in Table 2.

When the cell size of 64×64 was used to divide the image, the highest accuracy was achieved, and the average training time was low. Therefore, in the subsequent multi feature fusion, 64×64 cell size was selected to process the image. When a 256×256 image was subdivided into smaller units using 64×64 cell size, each cell corresponded to a 9-d histogram. The upper, lower, left, and right adjacent cells were regarded as a block of pixels. The feature dimension of each block was 4×9, i.e., 36 dimensions. Thus, the feature dimension of the whole image was 324 dimensions.

#### 2.3.2. Hu Moment Invariants Features

The shape information of corn and weed leaves can be used as the basis of classification, and their shape information can be expressed by some parameters to a certain extent. Hu invariant moment, which was proposed by Hu in 1962 [27], is a feature descriptor with translation and rotation. In the discrete case, Chen [28] improved it. Using Chen’s improved moment invariant algorithm, the positive and negative sample set images were grayed, and seven Hu moment invariants parameters and the eighth moment invariant parameter [29] were obtained to extract the shape information of positive and negative samples, which made the extracted leaf shape information more comprehensive. The calculation methods of the first seven feature parameters of Hu moment were mentioned in previous literature and were not repeated here. The formula of the eighth parameter is shown in Formula (1), as follows:

(1) $$\phi_{8}=2\eta_{11}\left[{\left(\eta_{30}+\eta_{12}\right)}^{2}-{\left(\eta_{03}+\eta_{21}\right)}^{2}\right]-2\left(\eta_{20}-\eta_{02}\right)\left(\eta_{30}+\eta_{12}\right)\left(\eta_{21}+\eta_{03}\right)$$

#### 2.3.3. Rotation Invariant LBP Features

**Figure 6:**
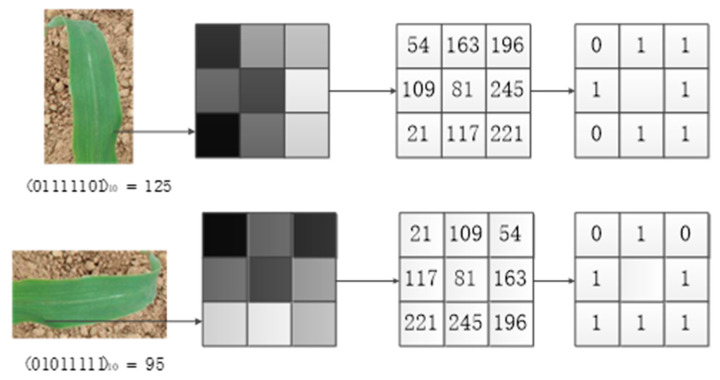
Schematic diagram of rotation invariant local binary pattern (RotLBP).

LBP is a texture structure that can reflect the microstructure between pixels. It is widely used in plant leaf image classification and achieves high classification accuracy. The improved rotation invariant local binary pattern (RotLBP) features have the advantages of simple calculation, rotation invariance, and gray level shift invariance [30]. After the image was rotated, the adjacent points corresponding to the center pixel changed, thereby leading to changes in the generated binary sequence. Moreover, the decimal value differed. To get a consistent LBP value, the smallest value among all binary sequences was selected as the rotation invariant LBP value of this pixel, as shown in Figure 6. When the original leaves rotated in different directions, the LBP values of the center pixel were 125, 250, 245, 235, 215, 175, 95, and 190. So, the rotation invariant LBP feature value of the center pixel was 95.

The principle of cell size selection method was consistent with that of the HOG feature. Table 3 is shown for details. The highest accuracy was obtained when 64×64 cell size was used. After dividing each cell into small areas, the rotation invariant LBP value of a pixel in each cell was calculated according to the 3×3 neighborhood. The feature dimension of the whole image was 160 dimensions (4×4×10=160).

#### 2.3.4. Gabor Features

Gabor filter can obtain optimal local features in both frequency domain and spatial domain. Thus, it has excellent directional selectivity and spatial locality and can be used for plant leaf texture detection [10]. In this paper, Gabor filter with 5 scales and 8 directions was used to filter the leaf image, and 40 sub images were obtained. Each sub image was divided into 3 × 3 sub blocks, and the Gabor feature of 360 (5 × 8 × 3 × 3) dimensional leaf images were obtained. The effect of Gabor filter was to let the information of a certain frequency band pass through it, and the rest of the information was filtered out. When extracting feature images for convolution filtering, the size of filter frequency domain window affects the bandwidth of filter in the frequency domain, and the size of filter convolution template affects the window of filter convolution template [31].

#### 2.3.5. Gray Level Co-Occurrence Matrix Features

GLCM can reflect the spatial correlation of gray values at any two points in the image. Six statistics of GLCM, namely, energy, contrast, correlation, sum entropy of co-occurrence matrix, entropy, and inverse difference moment, can reflect the texture features of the leaf image. The specific calculation formula is in reference [26].

**Figure 7:**
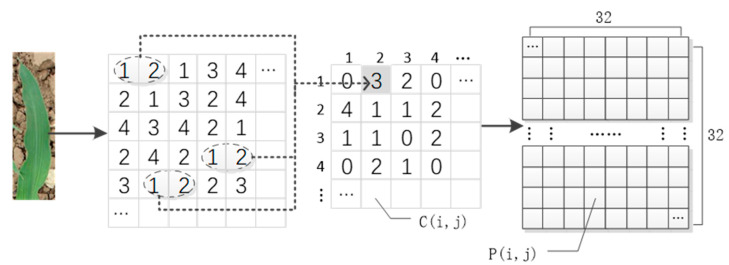
Generation process of gray level co-occurrence matrix.

Among moments, the second moment reflects the uniformity of gray distribution and texture thickness. It is also called energy, which is the sum of squares of each element of GLCM. A larger value indicates that the texture is thicker, and the energy is greater. Correlation is used to measure the similarity of gray symbiosis matrix in row or column direction, such as vertical texture, and the value in the c= 90° direction is greater than that in other directions. Entropy is the measure of image information. When the image is not textured, the value is 0. The maximum value is full texture. Contrast indicates the sharpness of the image. A deep texture groove indicates a clearer effect and a greater value. The inverse moment reflects the homogeneity of image texture. A large value of diagonal elements of gray symbiosis matrix leads to a greater value. The large value indicated the lack of change between different regions of image texture, and the local was very uniform. The correlation between these statistics was small, and the resolution effect was good, which represented the texture information of the image well. When extracting image features, we determined the sampling displacement vector d= (1,0), i.e., GLCM in the direction of 0°. In addition, the 45°, 90°, and 135° directions were selected, and the accuracy was 88.80%, 86.25%, 77.75%, and 76.5%, respectively. GLCM in the direction of 0° was the best. According to the d distribution, we calculated the numbers of gray levels i and j, respectively. Figure 7 shows an example for the calculation process of P(i,j). We calculated the matrix characteristics of gray level co-occurrence of the original image according to this statistic as follows:

(2) $$P\left(i,j\right)=\frac{C\left(i,j\right)}{N}$$

where C(i,j) represents the number of simultaneous occurrences of pixel pairs with gray value i and gray value j. P(i,j) is the probability of the occurrence of the pixel pair. N represents the total number of times that all pixel pairs with a distance of 1 in the 0° direction appear. The quantization level of gray level is 64.

#### 2.3.6. Gray Level-Gradient Co-Occurrence Matrix Features

GGCM was proposed on the basis of GLCM. In addition to the gray information of the image, the gradient information of the image was also considered. The gradient information of the image was obtained by image derivation, which reflected the gray level change on the image edge, especially in the boundary, edge, and other parts of the image with obvious change of gray value [32].

GLCM texture features used 15 statistical values [33], namely, small gradient advantage, large gradient advantage, gray-scale distribution nonuniformity, gradient distribution nonuniformity, energy, gray-scale average, gradient average, gray-scale mean square error, correlation, gray-scale entropy, gradient entropy, mixing entropy, inertia, and deficit moment [33]. Reference [33] presented the specific calculation method, which was not repeated here. Obviously, its characteristic dimension was 15.

### 2.4. Identification and Detection Process of Weeds and Corn Seedlings

The operating environment of all experiments in this paper was MATLAB 2018b, and the processor had the following features: core i7-9750H@2.60GHz X 12, GeForce RTX 2060 with 16 GB memory, and 6 GB of video memory. Window10 was the operating system.

#### 2.4.1. Training and Validation of Leaves Dataset

The specific experimental steps were as follows:

(1) First, the preprocessed positive and negative image samples were read successively. Each contained 1000 samples.

(2) The features of the positive and negative samples were extracted in sequence. The features were fused by multiple features. After extracting each feature separately, all the data were concatenated and stored separately. The specific single feature and various feature fusion methods are shown in Table 4. A total of 24 sets of experiments were conducted.

(3) The feature data of the positive and negative samples were processed by the feature dimensionality reduction method. The PCA method was used here. Then, the dimensionality-reduced positive and negative sample principal component data and the corresponding principal component coefficient matrix were saved. The principal component coefficient matrix is a matrix of p×p, p is the data dimension after feature dimensionality reduction.

(4) The acquired positive and negative sample feature data were integrated into a table, and a label value was added to distinguish positive and negative samples, where positive sample was labeled as 1, and the negative sample was labeled as −1. 

(5) The order of data in the table was disrupted. We randomly used 70% positive and negative sample data into the SVM classifier for training and saved the trained model for later testing.

(6) We use the remaining 30% of the sample data for verification to obtain the accuracy of the model. To show the results, a 2D scatter diagram was used to represent the positive and negative sample points. The first and second eigenvalues of the sample were defined as the x-coordinate and y-coordinate, respectively.

#### 2.4.2. Test of Actual Field Dataset at Corn Seedling Stage

A total of 400 actual corn seedling field images under different conditions were used for actual testing of weeds and corn seedlings detection performance based on the proposed method. Most of the images contained 2 to 3 corn seedlings and 3 to 4 or more weeds. These 400 actual field images contained more than 1000 corn seedlings and more than 1600 weeds. The test details are as follows:

First, convert the corn field image from the RGB color space to the Lab color space. According to the color information of channel a, they could be divided into foreground (weeds and corn seedlings) and background (soil). On this basis, two clustering regions were obtained by using the k-means clustering algorithm. To extract the features of the object region in the next step, the connected component analysis method was used to eliminate the clusters with too small an area and label the external rectangle of the clustering area where the plants are located. Finally, the relevant features of the target block were extracted, and the positive and negative principal component coefficient matrixes obtained in step 3 with the corresponding positive and negative sample data were multiplied during dimensionality reduction to obtain the dimensionality reduction data under the same mapping relationship. Then, the recognition model with the highest average recognition rate of weeds and corn obtained in step 6 was used to determine the type of each target area (corn or weed), and the result and target area were marked on the original image. The position of corn was marked with a yellow rectangle, and the position of weeds was marked with a red rectangle.

## 3. Results

### 3.1. Experiment on Leaves DataSet

In the experiment, 18 kinds of fusion strategies features were used besides the 6 single features, including HOG, rotation-invariant LBP, Hu invariant moments, Gabor, GLCM, and GGCM. For the robustness of the algorithm, the image data used in the verification was 30% of images randomly selected in the sample set.

Each group of experiments was conducted 10 times. Then, the accuracy of each experiment and the average of the ten times were recorded. Specifically, for the small constructed dataset that included corn leaves and weeds, the experimental results are shown in Table 5 and sorted in descending order of average accuracy. The first 6 groups are single feature group experiments, and the last 18 groups are multiple feature fusion group experiments. The training time was the time for training 1400 positive and negative samples with parallel operation. The last column in the table represents the number of observations that can be made per second during the verification. The time taken to extract the features was not included. The parameters contained in each set of observations were the feature parameters extracted by a target. The accuracy was used to correctly identify the proportion of corn seedlings and weeds in the total target quantity. The formula to define the classification accuracy is as follows:

(3) $$Accuracy=\frac{TP+TN}{TP+FP+FN+TN}$$

where *TP* is defined as corn seedling detected as corn seedling, *FP* is defined as weed detected as corn seedling, *FN* is defined as corn seedling detected as weed. *TN* is defined as weed detected as weed.

**Figure 8:**
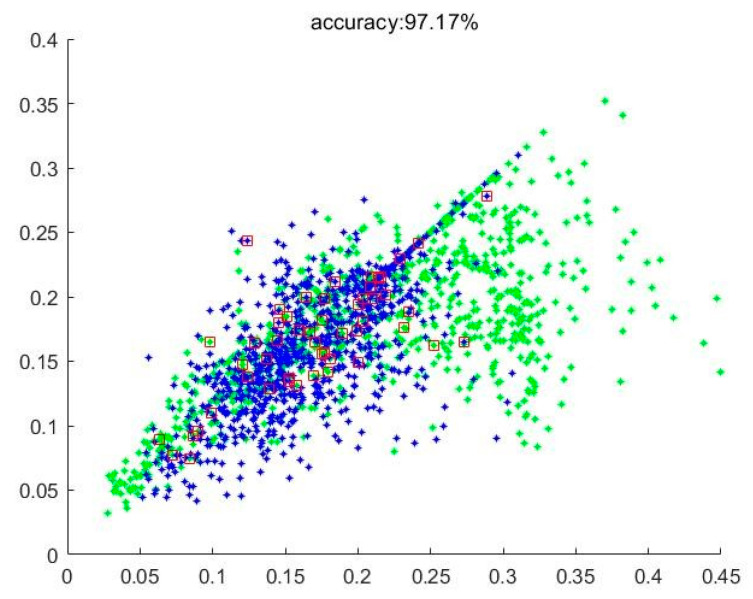
Scatter plot of test results using RotLBP and gray level-gradient co-occurrence matrix (GGCM) fusion features.

Figure 8 shows the result of verification using RotLBP and GGCM fusion features, in which corn is marked as green, weeds are marked as blue, and the detection error data are marked with a red box. We set different values of p, repeated the above process, and recorded the accuracy of related experiments. Finally, the recognition model with the highest accuracy was retained.

Observing the experimental results, the recognition rates of the seventh, eighth, and ninth fusion strategies in Table 5 were all higher than 96%. When the rotation-invariant LBP features and GGCM features were fused, the verification experiment achieved the highest average accuracy. The training and testing times were lower than those of the other two groups, and the accuracy and real-time performance were most in line with actual requirements.

### 3.2. Actual Field Image Test

In addition to randomly selecting 30% of the images from the constructed positive and negative image dataset for verification, 400 actual images of corn fields were also tested. The verification results showed that the highest experimental accuracy can be obtained when GGCM was fused with the rotating invariant LBP feature descriptor. To verify the effectiveness of GGCM and rotation in variant LBP fusion strategies in the detection of corn seedlings and weeds, we conducted a test.

**Figure 9:**
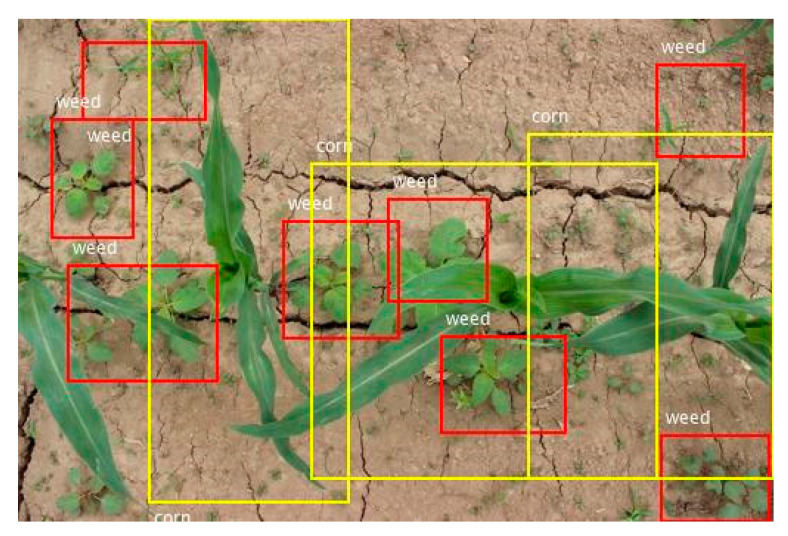
Experimental effect Ⅰ.

**Figure 10:**
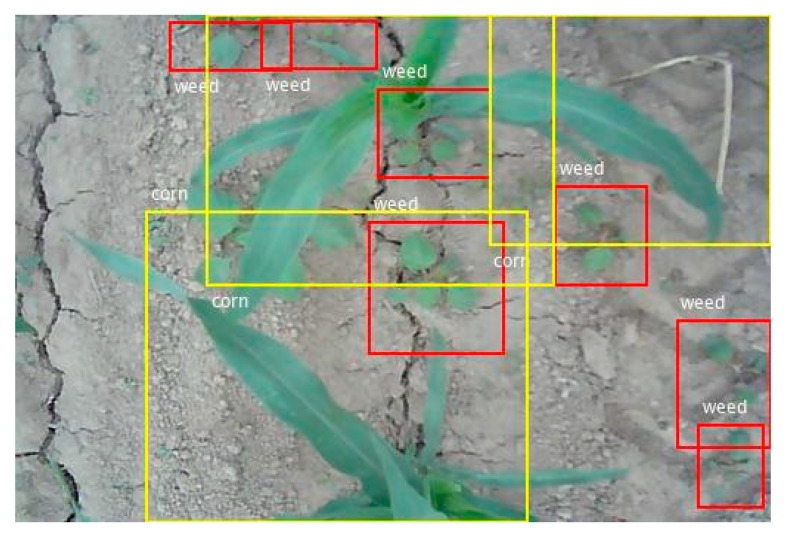
Experimental effect Ⅱ.

**Figure 11:**
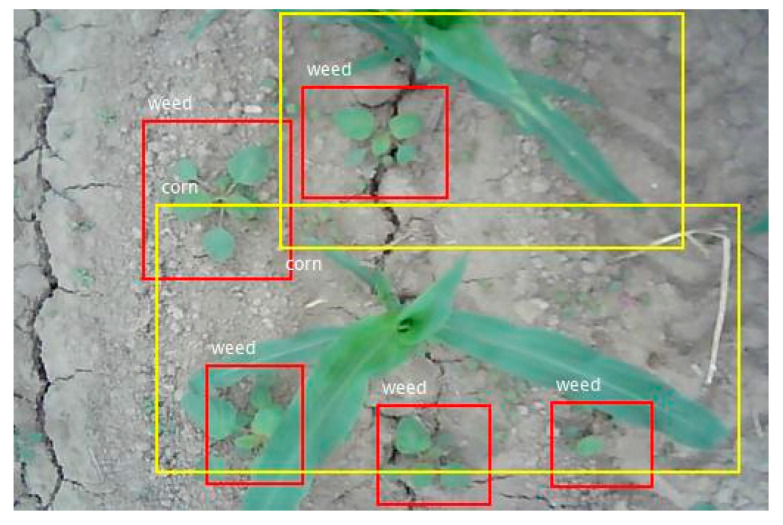
Experimental effect Ⅲ.

There are three of the actual detection results, as shown in Figure 9, Figure 10 and Figure 11. In the figures, the red rectangle is the detected weed region, and the yellow rectangle is the detected corn seedling region. Actual tested corn field image background is very complex, with some factors, such as partial overlap, object occlusion, and soil agglomerate, which put forward higher requirements for the detection algorithm. However, the experimental results were still very accurate and achieved the experimental purpose.

## 4. Discussions

As presented in Figure 9, Figure 10 and Figure 11, the weed and crop target detection results in the actual image showed that the system can accurately identify and detect corn seedlings and weeds in the image. The weed area detection in each image was very accurate. It provided accurate position information of the weeds and corn seedling to the spraying herbicide machine for precise spraying or to the fertilization machine for precise fertilizing.

**Figure 12:**
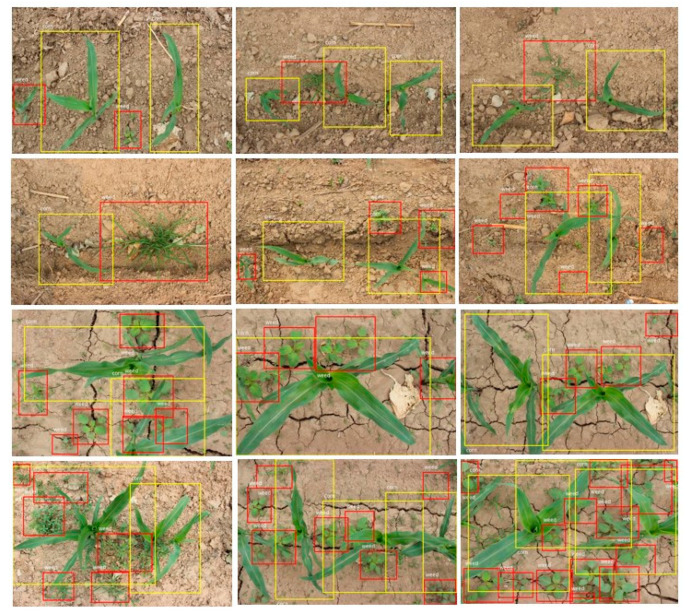
Experimental effect IV.

Figure 12 displays the detection results of a number of field images that are corn seedling plants. The detection performance of corn seedlings and weeds was very accurate.

In Figure 9, the leftmost corn leaf was not detected. The factor that affected the accuracy was the incompleteness of the target in the image. When the integrity of the target appearing around the edge of the image was less than one-third, the system was not able to identify the target well. When applied to the actual environment, the input was an image video sequence. Thus, the incomplete target in a certain frame of image did not affect the experiment. In the next frame of image, the complete target was correctly identified.

In general, the algorithm proposed in this paper had a relatively high accuracy in identifying and detecting corn seedlings and weeds in actual field images. Although the accuracy of detection needed to be improved when the edge of the image was less than one-third of the plants, the actual test chart showed that the algorithm in this paper had a very good detection effect on the complex corn seedling weed area and can be used for the later stage. Precise spraying provided precise weed location information. In the process of spraying herbicide on actual farmland, spraying fertilizers on crops or spraying herbicides on weeds provided accurate location information.

According to Table 5, when the PCA method was used for HU invariant moments, gray-level co-occurrence matrices, and gray level-gradient matrices with fewer dimensions, the accuracy was reduced. When fusing all the features involved in this article (HOG, rotation invariant LBP, Gabor, GGCM, HU invariant moments, and other six features), compared with the combination of less than six feature types, the accuracy of the former experiment was not higher than that of the latter. For the two classification problems of corn seedlings and weeds, the number of fusion feature types was not better. When the number was large, a problem of feature redundancy was encountered, resulting in a lower recognition rate.

In addition, the various features used to classify weeds and corn seedlings were briefly analyzed as follows. According to the results in Table 5, the accuracy was the highest when rotation invariant LBP was fused with GGCM, and the overall accuracy rate was 97.50%. The rotation invariant LBP feature improves the rotation invariant on the traditional LBP. Thus, it is widely used in texture image classification. When using appropriate size cells, the rotation invariant LBP feature can be effectively used as the classification basis for corn and weeds. However, the GGCM combined both the gray level information of the image and the gradient information of the image and expressed more abundant image information. After combining these two features, leaf information was better utilized, and higher detection accuracy was obtained. On the contrary, the experimental accuracy was lowest when HU invariant moment was used. HU invariant moment was generally used to recognize large objects on the image, but it only uses low order moments. For images with small objects and complex texture features, the details of the image cannot be described. Thus, the accuracy is low. The leaf of corn seedling was larger than that of weed. The detection accuracy of corn seedling was higher when HU invariant moment was used, but the detection accuracy of fine weeds was greatly reduced. Several other features also played a role in weed detection. As the basic function of wavelet transform, Gabor function enhanced the features extracted from all directions and scales of the image. Image information can be obtained from multiple directions and scales and was conducive to the extraction of spatial frequency and local structure characteristics of corn and weeds in multiple directions. GLCM used the local information of the image, which represented the correlation of gray values between pixels in a specific image space position. However, it cannot make use of the global information of the image. HOG feature was not subject to rotation invariability, and the direction of corn seedling leaves and weeds leaves was random. The traditional HOG feature as the basis for distinguishing corn from weeds cannot solve this kind of problem. Compared with GLCM, GGCM with single feature increased the gradient information, but it also increased the experiment time.

In summary, when the rotation-invariant LBP feature and the GGCM were selected, the average accuracy was the highest, and the corn seedlings and weeds were better identified. The actual image detection results showed that the method in this paper was very accurate in detecting and marking the weed and corn seedling areas. It provided accurate location information for variable spraying or precise fertilizing.

## 5. Conclusions

To identify and detect corn seedlings and weeds in a corn seedling field, a small image dataset for algorithm testing was established, and a method for identifying and detecting corn seedlings and weeds based on multi-feature fusion and SVM was proposed. Experiments and discussions were conducted on the fusion methods and effects of different features. A feature fusion scheme with satisfactory detection effect and real-time performance was selected. Details were as follows:

(1) A corn seedling and weed leaf image dataset containing 2000 positive and negative image samples was established. This dataset can be used for the establishment and verification of corn seedling and weeds classification algorithm models. At the same time, the actual field image dataset at seedling stage was also established for the actual effect test of weed and corn seedling detection in the field. To detect weeds and corn seedlings in a field at corn seedling stages, a binary target detection and recognition method was proposed. This method effectively identified corn seedlings and weeds and provided information for subsequent field management, such as intelligent variable spraying, weeding, and precise fertilizing.

(2) Based on multiple feature extraction and fusion strategies, 18 fusion methods with six different features were proposed to be tested in the actual corn seedling stage field image dataset to find the optimal fusion strategy for weed and the corn seedling detection. The six features were HOG feature, rotation-invariant LBP feature, HU invariant moment, Gabor feature, gray level co-occurrence matrix, and gray level-gradient co-occurrence matrix.

(3) Considering the high dimensionality of the feature data of positive and negative samples, PCA was utilized by some experimental groups for dimensionality reduction, which effectively improved the accuracy of the experiment.

(4) In the case of six single features, the rotation invariant LBP feature corresponded to the highest classification accuracy. Based on the multi-feature fusion, 18 combinations were set. The highest recognition accuracy was achieved when the rotation-invariant LBP features and GGCM were combined. Compared with the other 17 multi-feature combinations, both the global information of the image and the local information of the image were used, and the experimental effect was the best. For the construction of the sample image dataset, the average recognition accuracy of corn seedlings and weeds reached 97.50%. For actual corn seedlings field test images, the method proposed in this paper was accurate for the detection and marking of weed and corn seedling areas and provided accurate location information of the weeds and corn seedling for the pesticide spraying robot or the precise fertilizing machine.

## Figures and Tables

**Table 1:** Number of data sets and image size.

Category	Training	Validation	Actual Field Test Image
Total number of images	1400	600	400
Positive sample/corn seedling	700	300	/
Negative sample/weed	700	300	/
Size	256 × 256	256 × 256	1024 × 600

**Table 2:** Experimental results of histogram of oriented gradient (HOG) feature with different cell sizes.

Cell Size	Dimension	Accuracy (%)	Average Training Time(s)
8 × 8	34,596	82.5	90.449
16 × 16	8100	81.3	29.648
32 × 32	1764	84.5	18.208
64 × 64	324	87.8	16.530
128 × 128	36	84.9	15.725

**Table 3:** Experimental results of RotLBP feature with different cell sizes.

Cell Size	Dimension	Accuracy (%)	Average Training Time(s)
8 × 8	10,240	89.4	41.157
16 × 16	2560	89.8	19.731
32 × 32	640	87.4	16.190
64 × 64	160	90.6	96.308
128 × 128	40	88.4	99.379

**Table 4:** Multi-feature fusion method and dimension before dimensionality reduction.

Single Feature		Multi Feature					
Feature	Dimension	Feature	Dimension	Feature	Dimension	Feature	Dimension
HOG	324	RotLBP+HOG	484	HOG+GLCM	330	RotLBP+HOG+Gabor	844
RotLBP	160	RotLBP+Gabor	520	Gabor+GGCM	375	GGCM+RotLBP+HOG	499
Gabor	360	RotLBP+GLCM	166	Gabor+HU	368	GLCM+ RotLBP +HOG	490
GLCM	6	GGCM+RotLBP	175	Gabor+GLCM	366	RotLBP+HOG+Gabor+GLCM	850
GGCM	15	RotLBP+HU	168	GGCM+HU	23	RotLBP+HOG+Gabor+GGCM	859
HU	8	HOG+Gabor	684	GGCM+HOG	339	RotLBP+HOG+ Gabor+HU+GGCM	867

**Table 5:** Test accuracy and time consumption of each group of experiments.

Num	Feature Combination	PCA Dimension /Initial Dimension	Average Accuracy (%)		Training Time (s)	Prediction Speed (obs/s)
			After PCA	Before PCA		
1	RotLBP	95/160	91.40	90.60	2.1701	10,000
2	GGCM	15/15	87.80	90.80	68.807	150,000
3	HOG	55/324	88.80	87.80	2.2017	12,000
4	GLCM	6/6	88.80	88.80	12.137	49,000
5	Gabor	360/360	84.60	87.40	7.5382	7500
6	HU	8/8	82.10	85.00	2.1648	20,000
7	GGCM+RotLBP	94/175	97.50	90.50	2.1903	10,000
8	HOG+RotLBP+GLCM+Gabor	66/850	97.00	90.50	4.2998	5600
9	HOG+RotLBP	102/484	96.60	88.80	3.247	7500
10	GLCM+RotLBP	105/166	95.90	90.80	2.1812	9700
11	RotLBP+HU	61/168	95.70	89.60	1.684	14,000
12	GGCM+RotLBP+HOG	99/499	95.30	89.50	3.259	7400
13	GLCM+RotLBP+HOG	179/490	95.20	89.60	4.2638	4800
14	HOG+RotLBP+Gabor+GGCM	86/859	94.90	89.30	5.3031	6300
15	RotLBP+HOG+Gabor+HU+GGCM	107/867	94.80	86.10	6.531	4900
16	GGCM+HOG	100/339	94.50	89.30	2.7609	7700
17	RotLBP+Gabor	60/520	94.40	89.00	3.153	8600
18	HOG+RotLBP+Gabor	84/844	93.90	89.80	5.2385	6200
19	GGCM+HU	20/23	92.50	89.60	2.1829	27,000
20	HOG+GLCM	35/330	92.00	87.50	2.1553	12,000
21	GGCM+Gabor	375/375	87.30	91.30	53.75	8900
22	HOG+Gabor	684/684	85.30	91.10	13.819	3600
23	Gabor+GLCM	30/366	90.50	88.50	9.6001	7100
24	Gabor+HU	368/368	85.70	90.20	8.2748	8100

## Data Availability

Not applicable.
